# A CRISPR/Cas12a-based portable platform for rapid detection of *Leptosphaeria maculans* in *Brassica* crops

**DOI:** 10.3389/fpls.2022.976510

**Published:** 2022-09-08

**Authors:** Rong Lei, Yuan Li, Limei Li, Jingyi Wang, Zhenhai Cui, Rui Ju, Li Jiang, Xiaoling Liao, Pinshan Wu, Xinyi Wang

**Affiliations:** ^1^Chinese Academy of Inspection and Quarantine, Beijing, China; ^2^Central Laboratory of Yongchuan Hospital, Chongqing Medical University, Chongqing, China; ^3^School of Life and Health, Dalian University, Dalian, China; ^4^College of Sciences, Shenyang Agricultural University, Shenyang, China; ^5^Key Laboratory of Soybean Molecular Design and Breeding, Northeast Institute of Geography and Agroecology, Chinese Academy of Sciences, Changchun, China; ^6^College of Metallurgy and Materials Engineering, Chongqing University of Science and Technology, Chongqing, China

**Keywords:** *L. maculans*, recombinase polymerase amplification (RPA), Cas12a, all-in-one chip, lateral flow assay, portable platform

## Abstract

Establishing a portable diagnostic method for identifying plant pathogens is essential to prevent the spread of plant disease, especially in field and customs inspections. *Leptosphaeria maculans* (*L. maculans*) is an aggressive fungus, which causes severe phoma stem canker of *Brassica napus*, responsible for major yield losses of oilseed rape worldwide. In this study, CRISPR/Cas12a-based detection system and recombinase polymerase amplification (RPA) technique were employed to develop a rapid and sensitive detection method for identifying *L. maculans*. The involved RPA pre-amplification and CRISPR/Cas12a cleavage confer considerable sensitivity and selectivity, which can be finished within 45 min with a LOD of 4.7 genomic DNA copies. This detection system was further developed to two portable platforms, i.e., one-pot lateral flow detection and all-in-one chip lateral flow assay (AOCLFA), which integrates the lyophilized recombinase polymerase amplification (RPA) reagents and lyophilized Cas12a cleavage reagents in one tube or chip. The developed portable platforms have flexible portability and simple operation for the detection of *L. maculans* from plant tissues in the field. The proposed portable suitcase containing the minimum equipment, regents, and AOCLFA meets the practical needs of rapid *on-site* disease screening of plant fungi, port quarantine, or pathogen spreading control.

## Introduction

Plant pathogenic fungi are well-known for damaging or destroying plants. Almost all plant pathogenic fungi live on their host plants, partly in the soil, or plant debris. Oilseed rape (*Brassica napus*) can suffer from the filamentous fungi, *i.e. Leptosphaeria maculans* (*L. maculans*) and *Leptosphaeria biglobosa (L. biglobosa)*, which cause stem canker disease (or blackleg) of oilseed rape (*B. napus*) (Rouxel and Balesdent, 2005; Fitt et al., 2006) and several subspecies of *B. oleracea* (Humphersonjones, 1985; Moreno-Rico et al., 2001; Dilmaghani et al., 2010, 2013; Piliponyte-Dzikiene et al., 2015). The blackleg disease has become endemic in most areas where canola is grown, causing severe yield loss annually in Australia or Canada (Van de Wouw et al., 2016).

*L. maculans* and *L. biglobosa* have similar life cycles, common growth, morphological characteristics, mode of initial infections, and ecological habitat preferences, and often co-exist in the same infected tissues of plants as observed in North America (West et al., 2001; Dilmaghani et al., 2009), Europe (West et al., 2002; Fitt et al., 2006), and Australia (Sprague et al., 2006; Vincenot et al., 2008). In North America, Europe, and Australia, *L. maculans* is the predominant cause of the blackleg disease (Dilmaghani et al., 2009); but in Eastern Asia (China, Japan and Korea), *L. biglobosa* is the major pathogen causing the blackleg disease in *Brassica* crops (Hong et al., 2009; Liu et al., 2014; Hao et al., 2015). *L. maculans* is a more aggressive pathogen, causing canker on stem base, while *L. biglobosa* is less aggressive, causing less damaging lesions higher up to the stem base (Zhou et al., 1999; West et al., 2001; Fitt et al., 2006). Because *L. maculans* shares the common ecological niche with *L. biglobosa*, it easily spreads to similar agro-climatic regions *via* inadvertent distribution of seeds and trade exchanges (Fitt et al., 2008; Liu et al., 2014). Therefore, preventive actions against the introduction of *L. maculans* are prioritized in these countries to stop the *L. maculans* occurrence (Zhang et al., 2013; Zhang and Fernando, 2018), thus *L. maculans* was listed as the quarantine pest in many countries. Therefore, accurate, efficient, and rapid detection of *L. maculans* in *Brassica* crops is pivotal to prevent its global spread.

Nucleic acid-based tests (NATs) offer advantages over immunoassays, cell culture, microscopy, and other techniques to become the gold standard for the accurate diagnosis of many infectious diseases involved in plants, animals, and humans (Rougemont et al., 2004; Zhou et al., 2011). The sequence variation in the internally transcribed spacer (ITS) region genes of Leptosphaeria is often used to discriminate the species using Polymerase Chain Reaction (PCR)-based assays (Bailey et al., 2009; Zhou et al., 2010; Ashfield et al., 2012), recombinase polymerase amplification (RPA) (Lei et al., 2019), and Loop-mediated Isothermal AMPlification (LAMP) (Jedryczka et al., 2013; Zhou et al., 2016), but the conserved sequences among closely related species or isolates are limited (Mahuku et al., 1996). CRISPR (Clustered Regularly Interspaced Short Palindromic Repeats) and CRISPR-associated enzyme (Cas) systems can recognize and cleave specific nucleic acid sequences (namely *cis*-cleavage), thus increasing further selectivity and specificity (Jinek et al., 2012; Cong et al., 2013; Kellner et al., 2019). Cas12a, an RNA-guided enzymes, recognizes dsDNA, and exhibits its non-specific cleavage activities for single-stranded DNA (ssDNA) upon activation (Chen et al., 2018; Swarts and Jinek, 2019). Combining the cleavage effect of Cas12a with isothermal amplification techniques has created a versatile rapid and specific platform, such as DETECTR (DNA Endonuclease Targeted CRISPR Trans Reporter) with fluorescence readout (Chen et al., 2018), Cas12VDet (Cas12a-based Visual Detection) in a one-pot reaction (Wang et al., 2019), lateral flow strips for visual readout (Gootenberg et al., 2017; Myhrvold et al., 2018), as well as colorimetric detection with AuNPs-DNA probe (Li et al., 2019; Yuan et al., 2020). In contrast, the visual readout mode is more suitable for *on-site* detection needs than the fluorescence signal requiring lab instruments.

Several one-pot visual fluorescence detection methods based on CRISPR/Cas have been developed to detect SARS-CoV-2 (Ding et al., 2020; Wang et al., 2021) or foodborne pathogens (Wang et al., 2020b). In this study, we aim to develop the lateral flow test-based portable platforms applicable in the field, involving one-pot lateral flow detection and all-in-one chip lateral flow assay (AOCLFA) for sensitive colorimetric detection of *L. maculans*, minimize the reliance on equipment and professional techniques, simplify the operations and avoid contamination. The portable platform integrates RPA amplification, CRISPR/Cas12a cleavage reaction, and lateral flow detection in a single tube or chip, enabling contamination-free detection. Both RPA reaction and CRISPR/Cas12a reaction can be performed in a potable incubator or thermal box. The potable platform involves only pipettes, Eppendorf tubes, lyophilized reagents, lateral flow strips, and a man-carried incubator, which can be easily packaged into a portable suitcase, making it feasible to fast screen *L. maculans* from suspected oilseed rape tissues at port or in the remote field.

## Materials and methods

### Materials and reagents

All primers, FQ reporter (quenched fluorescent DNA reporter FAM-TTATT-BHQ1) and LF reporter (lateral flow strip reporter, FAM-TTATT-Biotin, FAM-T_20_-Biotin) were synthesized by Sangon Biotech (Shanghai, China). The detailed sequences are listed in Supplementary Table 1. EnGen^Ⓡ^LbaCas12a and NEBuffer 3.1 (10×) (100 mM NaCl, 50 mM Tris-HCl, 10 mM MgCl_2_, 100 μg/ml) were purchased from New England Biolabs (MA, USA). The TwistAmp^Ⓡ^Basic kit for RPA and portable real-time fluorometer TwistDX T8 (TwistDX Co. UK) were purchased from TwistDx (Cambridge, United Kingdom). The lateral flow strips (Cat. No. JY0301) and fast nucleic acid release regents (Cat. No. BT0068) were from Tiosbio Biotechnology Co, Ltd. (Beijing, China). Plant DNA extraction kit with magnetic beads (DP342), RNase inhibitor (NG209), and DNase/RNase-free ddH_2_O were purchased from Tiangen (Beijing, China). Dithiothreitol (DTT) (Cat. No. 43816) was purchased from Sigma Aldrich (St. Louis, MO, USA). The Qubit^Ⓡ^2.0 Fluorometer and the Qubit^Ⓡ^dsDNA HS Assay kit were purchased from Invitrogen (Life technologies, Carlsbad, CA). PDMS prepolymer was purchased from Dow Corning (Midland, USA). The fluorescence quantifications were measured with a Roche LightCycler 480 (Roche, USA).

### Genomic DNA extraction from fungi hypha or plant materials

The hyphae or spores of cultured fungi were collected into a 1.5 ml tube, ground with quartz sand, and the genomic DNA was extracted using a plant DNA extraction kit. For plant materials, about 500 mg samples were ground into powder with a mortar and pestle after adding liquid nitrogen, transferred into a 1.5 ml tube, and the genomic DNA extraction was performed according to the manufacturer's instructions. The absorbed DNA on the magnetic beads was eluted using appropriate autoclaved distilled water, and stored at −20°C. The concentration of DNA was quantified using the Qubit^Ⓡ^2.0 Fluorometer and the Qubit^Ⓡ^dsDNA HS Assay kit following the manufacturer's instructions.

For the crude DNA extraction, fungal hyphae or plant tissues in a 2 ml Eppendorf tube were crushed with quartz sands and plastic pestle, then 100 μl of fast nucleic acid release reagents was added, and mixed by pipetting up and down 20 times. After 2 min of standing at room temperature, the supernatant was directly used as the template for RPA pre-amplification.

### Recombinase polymerase amplification

The primers for RPA reaction were designed according to the ITS sequence of *L. maculans* (GenBank No.: MG569595.1), *L. biglobosa* “brassicae” (GenBank No.: MH861367.1), and *L. biglobosa* “Canadensis” (GenBank No. FJ172238.1) using DNAMAN 8.0. The ITS genes of *L. maculans* and *L. biglobosa* were searched against nr/nt NCBI database in order to select the most conserved and specific molecular markers to discriminate *L. maculans* from *L. biglobosa* and other sequenced fungi. The primers were synthesized by Sangon Biotech (Shanghai, China).

A master mix containing 29.5 μl supplied rehydration buffer, 2.4 μl forward primer (10 μM), 2.4 μl reverse primer (10 μM), and 8.6 μl nuclease free water was added to an RPA TwistAmp^TM^basic pellet to dissolve the enzyme, followed by the addition of 2.5 μl magnesium acetate (280 mM) (Supplementary Table 2). After a brief vortex and spin down the reaction mixture, 10 μl of the reconstituted RPA reaction was added to new PCR tubes, then 1 μl purified DNA was added to each reaction and mixed by carefully pipetting up and down. One pellet could yield approximately four individual RPA reactions. The reactions were incubated at 37°C for 15–30 min.

### RPA/Cas12a-based fluorescent assay

The crRNA for the CRISPR detection system was designed according to the amplified ITS gene from *L. maculans*, and aligned to determine the highly conserved regions. The crRNAs for the CRISPR detection system was designed specifically for the amplification region and synthesized commercially by Sangon Biotech.

Genomic DNA extracted from fungi hyphae or diseased plant tissue was used as input for RPA reaction. The system of Cas12a-mediated fluorescent assay contained 0.125 μM of Cas12a, 0.25 μM of crRNA, 0.1 μM of FQ reporter, 10 U of recombinant RNase inhibitor, 2.5 mM DTT, 1×NEBuffer 3.1, and 1 μl of RPA pre-amplification buffer in 20 μl reaction volume (Supplementary Table 3). The reaction was performed at 37°C in a TwistDX T8 or LightCycler 480 instrument for 30 min. The fluorescence intensity was detected with λ_Ex/Em_ of 488/520 nm.

### RPA/Cas12a-based lateral flow assay

The Cas12a-mediated lateral flow assay was performed in a total of 20 μl reaction mixture containing 0.125 μM Cas12a, 0.25 μM crRNA, 0.5 μM LF reporter, 10 U recombinant RNase inhibitor, 2.5 mM DTT, 1×NEBuffer 3.1, and 2μl of RPA pre-amplification buffer (Supplementary Table 4). LF reporter was labeled with carboxyfluorescein (6-FAM) at the 5' and biotin at the 3' end of ssDNA, respectively. The 20 μl reaction mixture was incubated at 37°C with a portable metal incubator for 20 min, followed by the addition of 80 μl of ddH_2_O. Then a lateral flow strip was put into this solution for about 3 min. If the sample is positive, both the C line and the T line or only the T line (strong positive sample) will appear, while only the C line appears for the negative sample. The strip images were converted to 8-bit grayscale using ImageJ (https://imagej.nih.gov/ij/), to determine the gray value of T line and C line intensity.

### RPA/Cas12a-based one-pot detection

The RPA pre-amplification reagents (Table 1) were added to the TwistAmp^TM^ basic enzyme pellet, and 10 μl of the reconstituted RPA reaction buffer was distributed to a new PCR tube. One pellet yielded approximately four RPA reactions. If a larger number of reactions are needed, the master mix volume and pellets are scaled up accordingly. The CRISPR reaction reagents were prepared according to the components list in Table 1, then 4 μl of the mixture was added to the PCR tube lid. One microliter DNA sample was added to the bottom of the PCR tube and mixed by carefully pipetting up and down, then the PCR tubes were incubated at 37°C for 20 min. Subsequently, the CRISPR reagents pre-placed on the PCR tube lid were mixed with the RPA reaction buffer by inverting and centrifuging the tube, and incubated at 37°C for another 20 min. When the FQ reporter was used, the fluorescence signals were monitored *via* a portable fluorometer or real-time PCR fluorometer. When the LF reporter was used, a lateral flow strip was directly inserted into the reaction buffer after 85 μl water was added, and stand for about 3 min.

**Table 1:** One-pot detection and all-in-one chip detection.

**Reagent name**	**Components**	**One-pot detection**	**AOCLFA**
		**Volume (μl)**	**Volume (μl)**
RPA pre-amplification regent	Forward primer, 10 μM	2.4	2.4
	Reverse primer, 10 μM	2.4	2.4
	TwistAmp Rehydration Buffer	29.5	29.5
	LF reporter (10 μM)	4.0	4.0
	RNase free water	4.7	0
	MgAc_2_ (280 mM)	2.5	2.5
	Total	45.5 μl was added to TwistAmp^TM^ basic enzyme pellet (preparing 10 μl aliquots for 4 tubes)	40.8 μl was added to TwistAmp^TM^ basic enzyme pellet (preparing 13 μl aliquots to be lyophilized for 3 chips)
Cas12a reaction regent	NEBuffer 3.1 (10×)	7.2	5.0
	Cas12a (10 μM)	2.4	1.7
	DTT (0.1 M)	2.4	1.7
	RNase inhibitor (40 U/μl)	2.4	1.7
	crRNA (10 μM)	4.8	3.3
	Total	19.2 μl (preparing 4 μl aliquots on the tube lid for 4 tubes)	13.4 μl (preparing 4 μl aliquots to be lyophilized for 3 chips)

### Fabrication of all-in-one chip

The mold for the microfluidic chip was first designed using AutoCAD (Supplementary Figure 1) and fabricated by a 3D printer using high-temperature resin. Next, the PDMS prepolymer, a two-component mixture of base and the crosslinking agent was put on the resin mold, and cured at a moderately elevated temperature (52°C for 12 h) to replicate the desired feature. An inlet (Φ 0.5 mm) was drilled, and covered with a Parafilm. To make this RPA/CRISPR method applicable for *on-site* field assay, the 13 μl of RPA reagents and 4.0 μl of CRISPR reagents (Table 1) were pre-stored in the corresponding reaction chamber, and lyophilized in a benchtop freeze dry system. Finally, the chip was quickly attached with another PDMS layer with double-sided tape (DSMS, Deer Brand, Taiwan) on a super clean bench.

### RPA/Cas12a-based all-in-one chip detection

The all-in-one chip system included a self-contained microfluidic chip and a portable heater pad or box. First, 15 μl extracted DNA was introduced to the RPA reaction chamber of the chip, which was put in a portable heater box set at about 37°C. After a 20 min incubation, the RPA amplicons in the RPA reaction chamber were pushed to the CRISPR reaction chamber by air with the pipette. After a 20 min-incubation, 85 μl deionized water was added to drive the CRISPR reaction buffer into the lateral flow strip chamber, where a lateral flow strip was inserted. The visual test result could be directly observed with naked eyes from the lateral flow strip in the detection chamber.

### Specificity of RPA/Cas12a-based assay

To avoid the interference by other fungi which infect *Brassica napus/B. oleracea*, or have the similar morphology of fungal hyphae and spores, we selected three *Leptosphaeria* fungi and three *Diaporthe* fungi having similar fungi colony shapes as the specificity evaluation. Seven pathogens including target pathogen *L. maculans*, approximate species *L. biglobosa* “brassicae”, *L. biglobosa* canadensis”, *L. lindquistii, Diaporthe helianthin, Diaporthe phaseolorum var. caulivora*, and *Diaporthe phaseolorum var. meridionalis* were detected to determine the specificity of RPA/Cas12a-based fluorescent assay and lateral flow assay, respectively.

### Sensitivity of RPA/Cas12a-based assay

The genomic DNA of *L. maculans* hyphae and spores were 10-fold diluted from 2.21 ng/μl (4.7×10^4^ copies/μl) to 0.221 pg/μl (4.7 copies/μl), which were detected with RPA/Cas12a-based assay. In the two-step method, template DNA was amplified according to the section “Recombinase Polymerase Amplification,” then the RPA products were detected according to the section “RPA/Cas12a-based Fluorescent Assay” and “RPA/Cas12a-based Lateral Flow Assay,” respectively. In one-pot detection, template DNA was detected with FQ reporter or LF reporter according to Section “One-pot detection.” For all-in-one chip detection, 15 μl diluted DNA solution was added and detected as explained in Section “RPA/Cas12a-based All-in-on Chip Detection.”

### Application of the portable platforms for field sample assay

To check the applicability of the RPA/Cas12a-based assay for field samples, 200 mg *L. maculans* hypha was ground in liquid nitrogen and dispersed in 400 μl water, then 50 μl *L. maculans* hypha in water was added to 0.1 g healthy oilseed rape seeds, stems and cabbage leaves, respectively. The extracted DNA from healthy plant tissues and *L. maculans* hypha were compared using the RPA/Cas12a-based fluorescent assay and the AOCLFA. To evaluate the efficiency of fast nucleic acid release reagents for the plant tissues, 20, 40, and 60 μl *L. maculans* hypha in water were added to healthy cabbage leaves, and the DNA were extracted using plant DNA extraction kit and fast nucleic acid release reagents, respectively. Both extracted nucleic acids were detected using an RPA/Cas12a-based fluorescent assay. Furthermore, DNA extracted from sixteen unknown fungi hyphae were detected with the AOCLFA. These fungi DNA were also amplified with primer ITS1/ITS4, sequenced, and aligned using NCBI Blastn.

### Statistical analysis

Statistical analyses and Analysis of Variance (ANOVA) were performed with Microsoft Excel. RPA/Cas12a-based fluorescent experiments were repeated at least 3 times for each sample. All experimental results were shown as Mean ± SD unless otherwise stated.

## Results

### Design of RPA/Cas12a-based AOCLFA for nucleic acid detection

**Figure 1:**
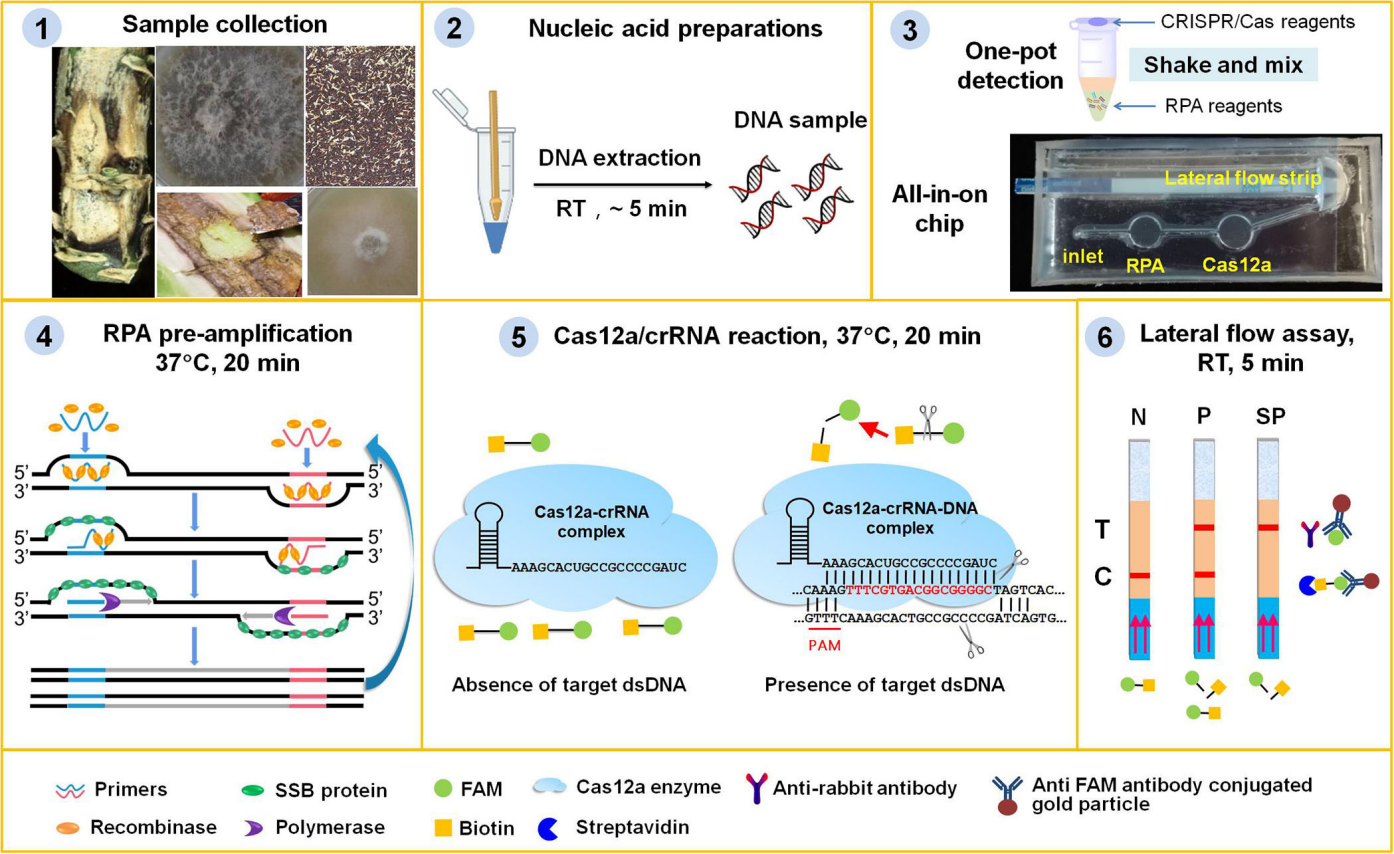
Principle of the RPA/Cas12a-based portable platform for the detection of plant fungi. (1) Plant tissues, such as stems with black canker symptoms, leaves with phoma leaf spotting, wizened oilseed rape seeds or residues with black spots, or fungi hyphae were collected from samples in field or at port. (2) Plant tissues or fungal hyphae were ground with quartz sands and plastic pestle to mud or powder, and the DNA was extracted with fast nucleic acid release reagents. (3) Lyophilized RPA pre-amplification reagents (containing the enzymes, primers, probe, and buffers), and Cas12a reagent mixture (containing crRNA, cas12a enzyme, and reaction buffer) were pre-stored in one tube or the chambers of the chip. (4) The fungi genomic DNA was pre-amplified with RPA enzymes and primers at 37°C for 20 min. (5) The RPA pre-amplification products were detected with CRISPR/Cas12a system at 37°C for 20 min. When the RPA products contained the target DNA, the binding of Cas12a/crRNA to target DNA would initiate its ability to digest reporter ssDNA with biotin and FAM group. (6) The intact ssDNA reporter was captured by the streptavidin on lateral flow strips to form control band, or else the cleaved FAM group binding with the FITC antibody conjugated gold particles moved to the test band. The lateral flow assay was complete within 5 min, and the bands could be immediately visually observed. N, negative sample; P, positive sample; SP, strong positive sample.

Lateral flow assay is one of the most convenient point-of-care testing (POCT) techniques and has been widely used for pathogen detection (Corstjens et al., 2001; Mao et al., 2009; Wang et al., 2020a). In this study, we adopted a paper-based lateral flow strip to develop two portable platforms, i.e., one-pot detection and an all-in-one chip lateral flow assay (AOCLFA) for the visual detection of *L. maculans*, which could reduce the reliance on equipment, simplify the operations, and avoid contamination. As illustrated in Figure 1, this portable platform integrated (i) RPA pre-amplification of sample DNA, (ii) sequence-specific recognition and non-specific cleavage by Cas12a/crRNA, and (iii) colorimetric readout of cleaved product from LF reporter. Both RPA pre-amplification reagents and Cas12a/crRNA reagents were pre-stored in one tube or different reaction chamber in one chip. For the negative result, only the control band would appear on the lateral flow strip, while both the test band and control band or only the test banc appeared in the presence of the positive sample. A portable incubator pad or thermal box with a mobile battery that can provide a temperature between 37 and 42°C was found to be suitable to meet the needs of both RPA and Cas12a reactions. By employing the fast plant DNA extraction procedure, the total time from sample preparation to results readout was <1 h. The minimum equipment required to operate the protocol included only pipettes, reagent tubes, a portable thermal box or pad, and lateral flow strips. All the equipment could be integrated into a portable suitcase. Therefore, the proposed method had great potential to enable *on-site* field assay of plant pathogen fungi outside of the laboratory.

### Performance of the RPA primers and Cas12a CrRNA for *L. maculans* detection

**Figure 2:**
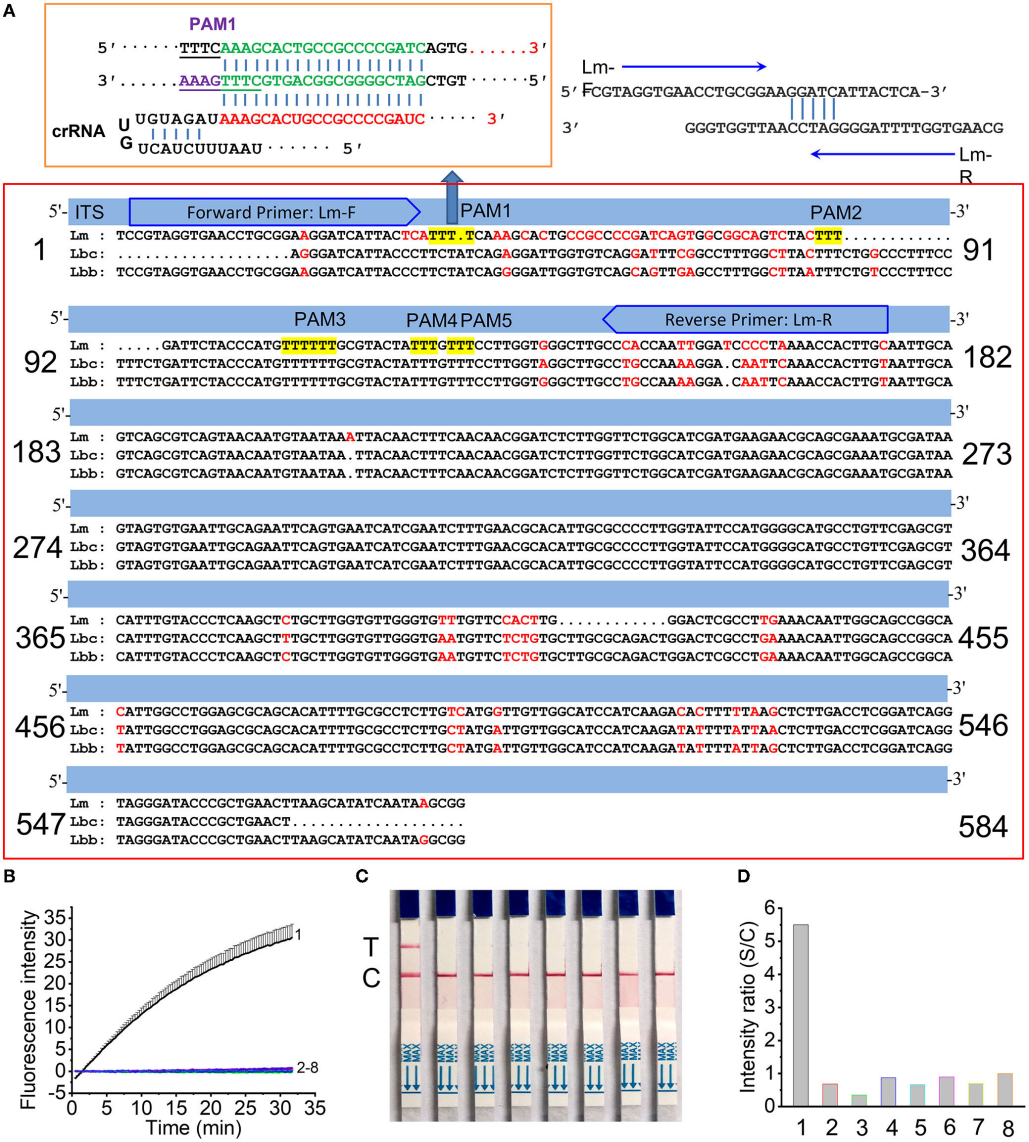
Specificity of RPA/Cas12a/crRNA for *L. maculans*. **(A)** Location of the primer set and crRNA in the ITS region of *L. maculans*. **(B,C)** Fluorescence signals **(B)** and lateral flow strips images **(C)** of Cas12a mediated detection of fungi genomic DNA. The data are presented as the mean ± SD from three independent experiments. **(D)** The gray value ratio of T line between the fungi genomic DNA and the negative control. 1, *Leptosphaeri maculans*; 2, *L. biglobosa* “brassicae”; 3, *L. biglobosa* “canadensis”; 4, *Diaporthe helianth*; 5, *Leptosphaeri lindquistii*; 6, *Diaporthe phaseolorum var. caulivora*; 7, *Diaporthe phaseolorum var. meridionalis;* 8, negative control.

The internally transcribed spacer (ITS) of *L. maculans* has limited sequence variance compared with its approximate species, i.e., *L. biglobosa* “brassicae” (Lbb) and *L. biglobosa* “canadensis” (Lbc) (Figure 2A). There are two regions (from 1 to 182 and from 365 to 546) having obvious variation between *L. maculans* and its approximate species. Since the region from 1 to 182 has more variance (56 bp) than the region from 365 to 546 (30 bp), primers Lm-F/Lm-R are located in the regions (1–182) were designed to produce amplicons of 154 bp. The protospacer adjacent motif (PAM) sequence of TTTN (Gootenberg et al., 2017; Chen et al., 2018) is required to recognize the target dsDNA by CRISPR/Cas12a. Among the five PAMs sites (TTTN) in the RPA target region of *L. maculans*, only the 20 bp after PAM1 site showed the maximum 9 nt difference with Lbc and 12nt difference with Lbb (Figure 2A). For the specificity of this crRNA designed from PAM1, the fluorescence signals using FL reporter as the ssDNA reporter indicated that only *L. maculans* genomic DNA generated high fluorescence signals (Figure 2B, line 1), but DNAs extracted from approximate species, such as *L. biglobosa* “brassicae,” *L. biglobosa* “canadensis,” *L. lindquistii, Diaporthe helianthi, Diaporthe phaseolorum var. caulivora* and *Diaporthe phaseolorum var. meridionalis* produced no obvious signals (Figure 2B, line 2–7). The lateral flow strip results also indicated that only *L. maculans* showed positive test lines using this Cas12a/crRNA detection system with LF reporter, and other allied species showed no positive lines (Figure 2C). The gray value ratio of the T line between the samples and the negative control indicated that *L. maculans* could produce detectable signals much stronger than other fungi (Figure 2D). Therefore, the designed primers and crRNA had good specificity for *L. maculans*.

### Optimization of RPA/Cas12a-based nucleic acid detection

**Figure 3:**
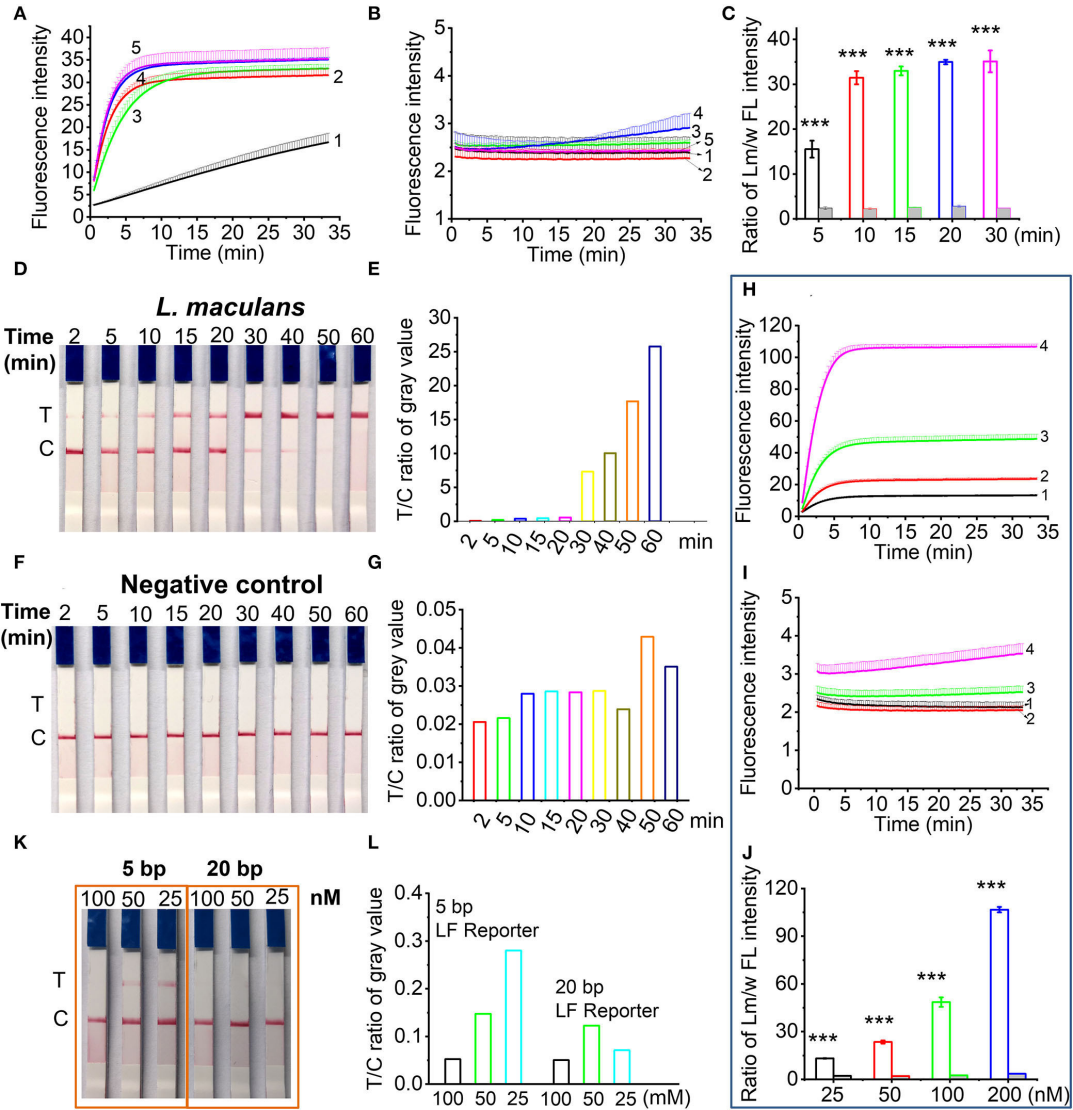
Optimization of RPA/Cas12a-based nucleic acid detection. **(A,B)** Real-time fluorescence signals of RPA products from *L. maculans* **(A)** and water **(B)** with different RPA reaction time. Line 1-5 indicated the RPA products with RPA time of 5, 10, 15, 20, and 30 min, respectively. **(C)** The ratio of fluorescence intensity (FI) between *L. maculans* and water at 30-min CRISPR reaction. **(D)** Lateral flow assay of *L. maculans* with different Cas12a/crRNA reaction time from 2 to 60 min, respectively. **(E)** The ratio of gray value between T line and C line of lateral flow strips in **(D)**. **(F)** Lateral flow assay of negative control (water). **(G)** The ratio of gray value between T line and C line of lateral flow strips in **(F)**. **(H,I)** Real-time fluorescence signals of RPA products of *L. maculans* **(H)** and water as the negative control **(I)** with different FL reporter concentrations. Line 1-4 indicated the concentration of FL reporter is 25, 50, 100, and 200 nM, respectively. **(J)** The ratio of fluorescence intensity between *L. maculans* and water at 30-min CRISPR reaction. **(K)** Lateral flow assay of 5 bp and 20 bp LF reporter with different concentrations. **(L)** The ratio of gray value between T line and C line of lateral flow strips in **(K)**. The data of ratio between Lm/w FL intensity are presented as the mean ± SD from three independent experiments. ^***^ indicates *p* < 0.001.

For RPA pre-amplification time, results (Figures 3A,B) indicated that shorter RPA pre-amplification time produced less amplicons, thus yielding lower CRISPR/Cas12a signals. There was no obvious signal difference when RPA time was more than 20 min, indicating that 20 min was enough for RPA pre-amplification (Figure 3C). Thus, 20 min was used as the optimized RPA pre-amplification time. For CRISPR/Cas12a reaction time, about 15 min was enough to produce a detectable test band for the RPA pre-amplification products of *L. maculans* (Figures 3D,E). However, prolonged incubation (e.g., 50 and 60 min) may increase non-specific products (Figures 3F,G). Therefore, 20–30 min was the optimized Cas12a/crRNA reaction time throughout the experiment.

The ssDNA reporter in CRISPR/Cas12a system was first evaluated using the FQ reporter involved in fluorescence detection. Four concentrations of FQ reporter, i.e., 25, 50, 100, and 200 nM were adopted in the Cas12a/crRNA detection system with the RPA pre-amplification products of *L. maculans* or water as the template. Results showed that FQ reporters with higher concentrations provided higher fluorescence signals (Figures 3H–J). Taking the cost into account, 100 nM of FQ reporters were used in the subsequent experiment. For lateral flow assay, it has been reported that 1 μM LF reporter (FAM-TTATT-Biotin) can avoid the false positive band (Lu et al., 2020). However, we found that both concentration and length of LF reporter affected the generation of the false-positive band. The results showed that a false-positive band appeared when a 5 bp LF reporter with a lower concentration than 100 nM was used; In contrast, when a 20 bp LF reporter (FAM-T_20_-Biotin) was used, false-positive bands could be avoided at lower concentrations (Figures 3K,L). Thus, the 20 bp ssDNA LF reporter with 100 nM was adopted in the following study.

### RPA/Cas12a-based one-pot detection

**Figure 4:**
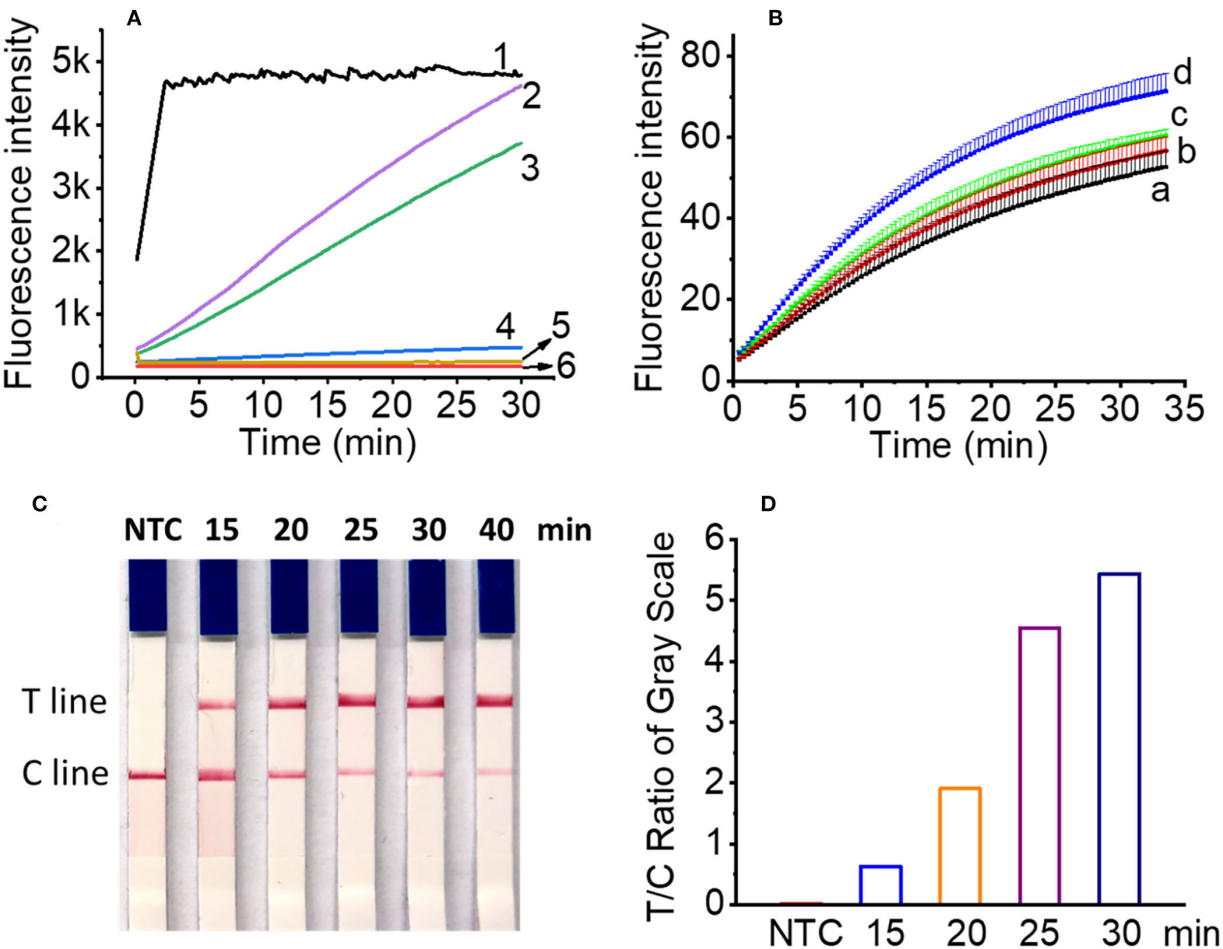
Optimization of one-pot reaction condition. **(A)** The real time fluorescence signals of two-step (line 1), optimized one-pot detection (line 2), FQ reporter in RPA buffer (line 3), all Cas12a reagents in RPA buffer except Cas12a enzyme and NEBuffer 3.1 (line 4), crRNA/DTT/RNase inhibitor in RPA buffer (line 5) for one-pot detection and negative control (line 6). **(B)** The real time fluorescence signals of RPA amplicons with reaction time of 15 min (line a), 20 min (line b), 25 min (line c), and 30 min (line d), respectively. **(C)** Lateral flow assay results of *L. maculans* RPA pre-amplification buffer with different Cas12a/crRNA reaction time in one-pot for 15 to 40 min. **(D)** The ratio of gray value between T line and C line of lateral flow strips in **(C)**.

To evaluate the possibility of all-in-one chip detection, we first performed the integration of RPA and Cas12a cleavage in a single tube. NEBuffer 3.1 was not included in RPA pre-amplification buffer, but on the tube lid to avoid the negative effect of Mg^2+^ in NEBuffer 3.1 on the RPA pre-amplification. Results showed that the addition of all the components except Cas12a enzyme and NEBuffer 3.1 to the RPA reaction produced no fluorescence signal (Figure 4A, line 4). To investigate which component affects the fluorescence signal, crRNA and FQ reporter were added separately to the RPA reaction, and the results showed that the inclusion of crRNA/DTT/RNase inhibitor in the RPA buffer produced no fluorescence signal (Figure 4A, line 5), while FQ reporter did not affect the generation of the fluorescence signal (Figure 4A, line 3). DTT and RNase inhibitors were jointly used to protect crRNA from degradation. Therefore, in one-pot detection, NEBuffer 3.1, Cas12a enzyme, DTT, RNase inhibitor, and crRNA were added to the tube lid or tube wall. After the RPA pre-amplification finished, the CRISPR/Cas12a reaction components were shaken into the RPA reaction buffer, and then the tube was incubated at 37°C for another 20 min.

For the RPA pre-amplification time involved in one-pot detection, four time points, i.e., 15, 20, 25, and 30 min were tested, and the results showed that although longer RPA pre-amplification produced higher fluorescence signals for the same sample (4.7 × 10^3^ copies DNA), the difference is not obvious, indicating 15 min was enough to produce RPA amplicons for one-pot detection (Figure 4B). For the lateral flow assay, 4 μl FQ reporter (2 μM) was replaced with 4 μl LF reporter (10 μM), and the cleaved LF reporter was detected by the lateral flow strip. To optimize the Cas12a reaction time, the products of four time points, i.e., 15, 20, 25, and 30 min were tested with lateral flow strips. The results showed that 15 min was enough to produce a detectable T line (Figures 4C,D), which was consistent with the two-step RPA/Cas12a experimental results (Figure 3D). The total time of RPA pre-amplification and Cas12a reaction was about 30 min.

### Sensitivity of RPA/Cas12a-based *L. maculans* detection

**Figure 5:**
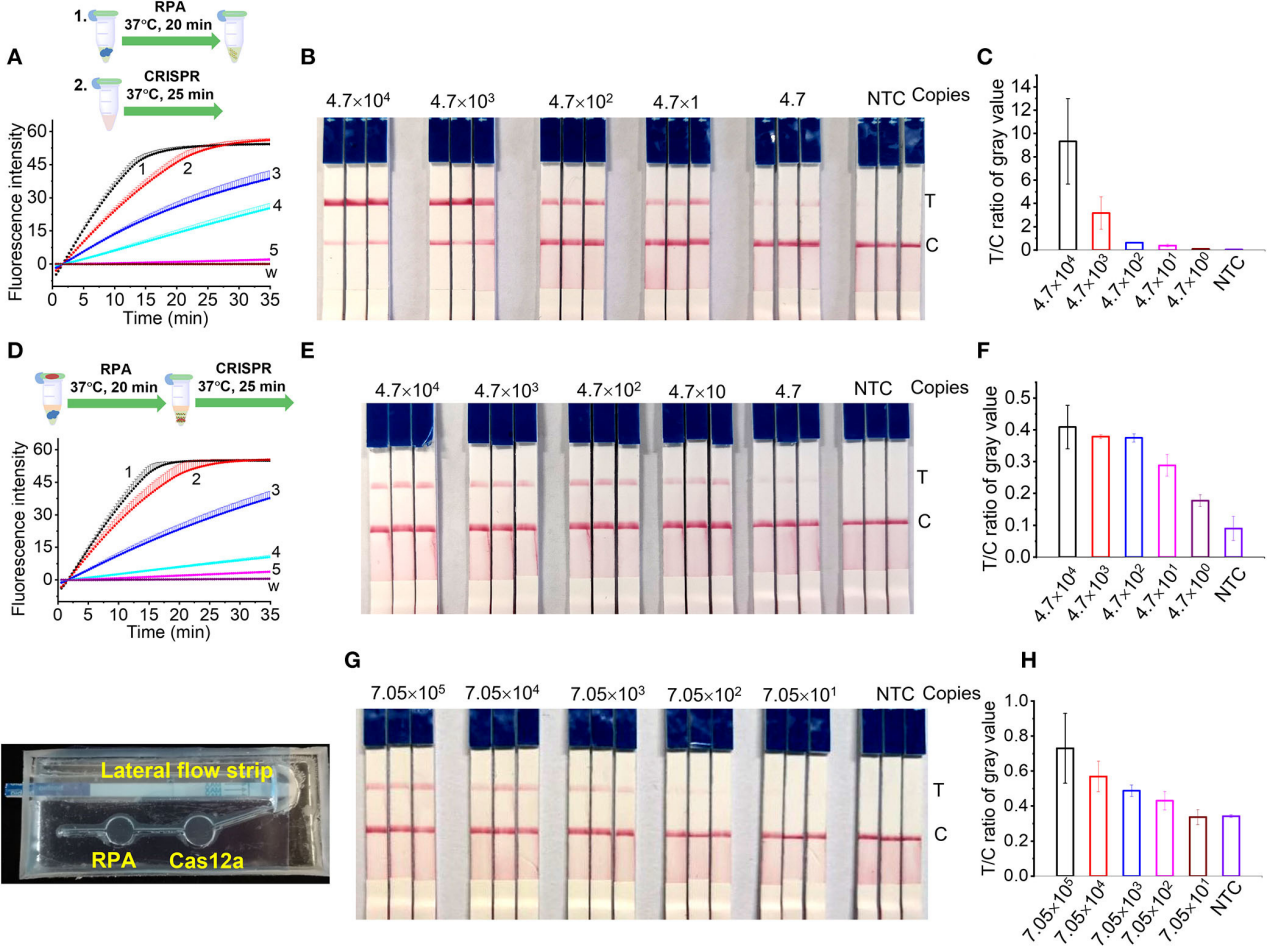
Sensitivity of RPA/CRISPR/Cas12a detection. **(A,B)** Two-step detection of *L. maculans* genomic DNA using fluorescence probe **(A)** and lateral flow strips **(B)**. **(C)** The ratio of gray value between T line and C line of lateral flow strips in **(B)**. **(D,E)** One-pot detection of *L. maculans* genomic DNA using fluorescence probe **(D)** and lateral flow strips **(E)**. **(F)** The ratio of gray value between T line and C line of lateral flow strips in **(E)**. Lines 1-5 indicated the genomic DNA copies. 1, 4.7 × 10^4^; 2, 4.7 × 10^3^; 3, 4.7 × 10^2^; 4, 4.7 × 10^1^; 5, 4.7. **(G)** Detection of AOCLFP. **(H)** The ratio of gray value between T line and C line of lateral flow strips in **(G)**.

The sensitivity of RPA/CRISPR/Cas12a detection was evaluated using the genomic DNA of *L. maculans* from 2.21 ng/μl (4.7 × 10^4^ copies/μl) to 0.221 pg/μl (4.7 copies/μl). For the two-step detection, the genomic DNA was pre-amplified with RPA for 20 min, then 2 μl pre-amplification products were added into the 18 μl of Cas12a/crRNA reaction buffer for another 20 min. Every reaction was repeated 3 times. The results showed that 0.221 pg (4.7 copies) of *L. maculans* could be detected with the developed Cas12a-mediated fluorescence detection (Figure 5A). This sensitivity is 100 times higher than that of fluorescent RPA and 10 times higher than that of real-time PCR (Lei et al., 2019), indicating that Cas12a-mediated detection combined with RPA had high sensitivity. For the two-step detection with lateral flow strips, the results showed that 2.21 pg (47 copies) of *L. maculans* was obviously detected, and 0.221 pg (4.7 copies) was weakly detected (Figure 5B). The gray value of the T line of 0.221 pg *L. maculans* DNA was higher than that of the negative control (Figure 5C). Thus, this sensitivity is 100 times higher than that of fluorescence RPA (Lei et al., 2019), indicating that RPA/CRISPR/Cas12a with lateral flow strips also had good sensitivity.

For one-pot detection, the same *L. maculans* genomic DNA samples as those in two-step detection were used to evaluate the sensitivity. The results showed that the sensitivity of the developed one-pot detection method using fluorescence reporter (Figure 5D) was the same as that of two-step detection (Figure 5A). Although the T lines of 2.21 ng and 0.221 ng *L. maculans* DNA were lighter than those in two-step detection, the T line of 0.221 pg *L. maculans* DNA was obviously stronger than that of negative control (Figure 5E). The gray value of the T line of 0.221 pg *L. maculans* DNA was higher than that of negative control (Figure 5F) indicating that one-pot lateral flow assay has the same sensitivity as that of two-step detection.

The sensitivity of AOCLFP was discounted compared with the two-step and one-pot detection. As shown in Figures 5G,H, the T line of 15 μL DNA (2.21 pg/μl) can be weakly detected, and the LOD was calculated as 705 copies. The lower sensitivity in AOCLFP may result from the inadequate mixing of injected DNA samples with the pre-stored lyophilized RPA reagents or CRISPR/Cas12a reagents in the chip chamber.

### Applicability of AOCLFA for field samples

**Figure 6:**
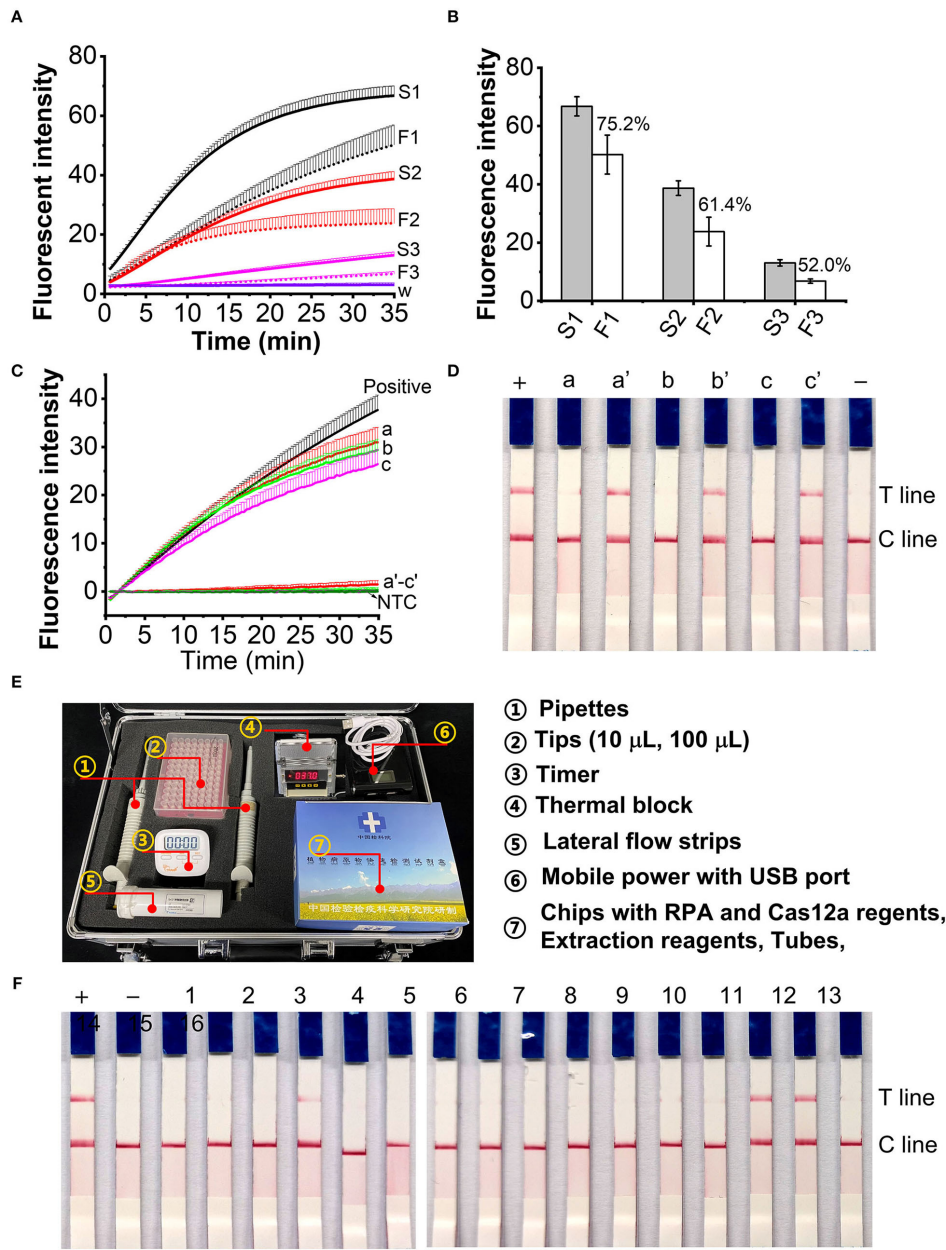
Application of the RPA/Cas12a-based assay for field samples. **(A)** Fluorescent signals comparison of extracted DNA from 60 μl (S1, F1), 40 μl (S2, F2), and 20 μl (S3, F3) using standard DNA extraction (S1, S2 and S3) and fast nucleic acid release reagents (F1, F2 and F3). **(B)** The ratio of fluorescence intensity (FI) at 30-min CRISPR reaction between S1-S3 and F1-F2, respectively. **(C,D)** Real time fluorescent signals **(C)** and lateral flow strips **(D)** of DNA extracted from plant tissues (a-c) and Lm hypha spiked plant tissues (a'-c'). a, a': cabbage leaves; b, b': oilseed rape stems; c, c': oilseed rape seeds. **(E)** Picture of portable suitcase. **(F)**, The lateral flow assay results of unknown fungi samples. –, negative control; +, positive control. Sample 1-16 was identified with ITS1/ITS4 PCR amplification and Sequencing (Supplementary Table 5).

The efficiency of crude DNA extraction is 75.2, 61.4, and 52.0% for 60, 40, and 20 μl *L. maculans* hyphae spiked plant tissues (Figures 6A,B). The extracted DNA samples from healthy plant tissues and *L. maculans* hypha spiked plant tissues were first determined with the RPA/Cas12a-based fluorescent assay. Results showed that there were no fluorescence signals for the healthy plant tissues (Figure 6C, line a–c), but *L. maculans* hypha spiked plant tissues had strong signals (Figure 6C, line a'–c'). The AOCLFP results showed that the healthy plant tissues showed no positive results, but the *L. maculans* hypha spiked plant tissues showed positive T lines (Figure 6D). To meet the field detection requirement, we developed a portable suitcase, which integrated pipettes, tubes, chips with lyophilized reagents, a portable thermal box, and lateral flow strips altogether (Figure 6E). This AOCLFA was applied to detect sixteen fungi samples, which were meanwhile identified with ITS1/ITS4 amplification and sequencing. The results showed that only *L. maculans* presented positive signals, and other fungi DNA presented negative signals (Figure 6F). The AOCLFA detection results of these 16 fungi were consistent with the NCBI Blastn results of the sequencing (Supplementary Table 5).

## Discussion

Plant pathogenic fungi are very harmful to plant growth and agricultural product quality. Blackleg disease caused by *L. maculans* is the most serious disease of *B. napus* (canola, oilseed rape) and *B. oleracea* (Piliponyte-Dzikiene et al., 2015) grown worldwide (Fitt et al., 2008). Due to its potential risk as a seed contaminant, the pathogen may lead to trade barriers in international canola seed exports. The highly-virulent *L. maculans* produce the phytotoxin sirodesmin PL, while the weakly-virulent *L. biglobosa* does not produce phytotoxin (Balesdent et al., 1998). *L. maculans* and *L. biglobosa* are pair of sibling species with similar symptoms and often co-exist in the same infected tissues, which brings considerable interference to the identification of pathogens. Therefore, identifying *L. maculans* in plant tissues with blackleg symptoms or in canola seeds is pivotal to preventing the spreading of *L. maculans* in the field or at port. Although we developed a rapid detection of *L. maculans* using a RPA-based fluorescence detection method (Lei et al., 2019), we found that it is not convenient to carry a fluorescence detector with a high-power mobile battery and perform the experiments in the field. Thus, we turned our attention to the lateral flow assay, one of the most convenient point-of-care testing (POCT) techniques, which has been widely used for pathogen detection (Corstjens et al., 2001; Mao et al., 2009; Wang et al., 2020a). Under the blessing of the CRISPR/crRNA system, the drawbacks of false positives in lateral flow detection have been greatly alleviated. The CRISPR system including many different Cas proteins with nuclease activity has been adopted to detect nucleic acids (Gootenberg et al., 2018). Amongst, Cas12a has the ability of dsDNA recognition and non-specific ssDNA collateral cleavage (Chen et al., 2018). The ssDNA can be modified with small molecular groups, such as Fam, Biotin, BHQ1, et al., so the released Fam or Biotin can be detected using a fluorimeter or lateral flow assay. In this study, we employed Fam-T_20_-Biotin as the ssDNA reporter of combined RPA amplification and Cas12a-based detection to establish a portable all-in-one chip flow lateral assay for *L. maculans*, in order to reduce the reliance on equipment, simplify the operations and avoid contamination. The minimum equipment required to operate the protocol includes only pipettes, reagent tubes, a portable thermal block, and lateral flow strips. All the equipment can be integrated into a 42 × 30 × 15 cm suitcase (Figure 6E). The proposed method has great potential to enable *on-site* field assay of plant pathogen fungi outside of the laboratory.

The internally transcribed spacer (ITS) genes of fungi are conserved among closely related species or isolates (Fajarningsih, 2016), so enormous reference sequences have existed in the NCBI sequence database, such as Genbank, EMBL, etc. (Schoch et al., 2012). Although sequence variation in the ITS region has been often chosen as molecular targets to identify fungi (Bellemain et al., 2010; Das and Deb, 2015), ITS region genes among closely related species or isolates have scant sequence variation, which makes it difficult to develop highly sensitive *on-site* detection methods only based on isothermal amplification techniques (Lei et al., 2019). Due to the recognition of crRNA to the target dsDNA, the combined RPA and CRISPR/Cas12a system has better specificity and sensitivity than single RPA (Roy and Kirchner, 2000; Chen et al., 2018; Aman et al., 2020). We designed the RPA primes to produce amplicons of 152 bp, which was close to the optimized amplicon size between 80 and 140 bp for CRISPR/Cas12a-based nuclei acid detection (Kellner et al., 2019). Even though there are five PAMs sites (TTTN or NAAA) in the RPA amplicons, only PAM1 is appropriate for the design of crRNA, since the subsequent dsDNA sequences had the maximum variation with the *L. biglobosa* (Figure 2A). The results confirmed that this crRNA can specifically detect *L. maculans*.

In the combined RPA/CRISPR/Cas12a strategy, some key parameters, including ssDNA reporter concentration, RPA pre-amplification time, and Cas12a/crRNA cleavage reaction time are the important factors to determine the fluorescence readout or lateral flow assay. The real time fluorescence curves with FQ reporter indicated that higher concentration generated higher intensity, but also increased background interference. With the comprehensive consideration of sensitivity and cost, 100 nM of FQ reporter in the final detection buffer was adopted. However, fluorescence detection demanded a device to excite the released fluorophore and specific filters to measure the emitted light, which required black background, which makes it not feasible for *on-site* detection in field. Combining RPA and lateral flow assay could replace the instrument-dependent PCR amplification technology and fluorescence detection method. One important issue in lateral flow assay is avoiding the false positive bands to improve the accuracy and sensitivity. A low concentration of LF reporter easily resulted in the false positive band, since a portion of nanoparticles did not bind to the FAM-Biotin ssDNA reporter, kept moving, and reached the test band to form the potential false-positive result. One micromole LF reporter has been used in the lateral flow assay to prevent the false positive band (Lu et al., 2020). However, we found that the length of ssDNA also affects the false positive band. Compared with 5 bp LF reporter, 20 bp LF reporter at lower concentration did not produce a false positive band (Figures 3K,L). We deduced that longer ssDNA has better purity than shorter ssDNA sequences, and thus free FAM can be effectively avoided.

Up to now, several detection methods have been developed for *L. maculans*, including PCR-based methods (Long et al., 2017), qPCR (Zhou et al., 2011), LAMP (Zhou et al., 2016; Du et al., 2021), and RPA (Lei et al., 2019) (Table 2). Nested PCR offers the best sensitivity, but it is the most time-consuming and needs instruments with thermal cycling and electrophoresis instrument (Song et al., 2016). LAMP, in spite of being one of the widely used isothermal amplification techniques, requires high temperatures up to 65°C (Zhou et al., 2016; Du et al., 2021), which is not well compatible with field detection requirements. In contrast, both RPA and CRISPR/Cas12a detection systems run around 37°C, take less time, and are more practicable for the application in rapid *on-site* detection. Furthermore, various one-step strategies based on CRISPR/Cas12a coupled with isothermal amplification techniques have been developed with the advantages of flexibility and cross-contamination prevention (Ding et al., 2020; Wang et al., 2020b, 2021; Yin et al., 2020). At the signal output, the sensitive fluorescence visualization readout has suffered from interference from the high background, as well as specific excitation light and filter. The lateral flow assay have several advantages: visualization with naked eyes, portability, simple operation, high sensitivity, and specificity (Broughton et al., 2020), which is a perfect visual signal readout after resolving the high incidence of false positives. A recent application is the integration of RT-RPA and CRISPR/Cas systems into a closed microfluidic platform for instrument-free point-of-care (POCT) diagnosis of SARS-CoV-2, which prepared the RT-RPA reaction mixture of the chip (Li et al., 2022). In the developed AOCLFA in this study, the lyophilized RPA reagents and CRISPR/Cas12a reagents were pre-stored in one chip, and the extracted DNA sample could be directly injected into the RPA reaction chamber, therefore, the operation and transportation of reagents can be greatly simplified. Although chip processing and batch assembly processes need further improvement, the proposed AOCLFA platform showed huge potential for the efficient, accurate, simple, and field detection of plant pathogenic fungi.

**Table 2:** Comparison of different detection methods for *L. maculans*.

**Method**	**Time**	**Assay** **type**	**Main** **instrument**	**LOD**	**Advantages**	**Disadvantages**	**References**
Nested PCR	4h	DNA	PCR thermocycler	0.01 pg/μl	High sensitivity	Time consuming, requiring complex instruments	Song et al., 2016
DPO-PCR	3h	DNA	PCR thermocycler	10 pg/μl	High specificity	Time consuming, requiring complex instruments	Long et al., 2017
qPCR	2~4 h	DNA	Real-time PCR instrument	4 pg	High specificity	Complex operation, expensive cost	Zhou et al., 2011
LAMP-LFD	2 h	DNA	65°C, PCR thermocycler	0.114 pg/μl	High sensitivity	High temperature, false-positive risk	Zhou et al., 2016
LAMP-fluorescence	~2 h	DNA	65°C, PCR thermocycler	0.696 pg	High sensitivity	High temperature, false-positive risk	Du et al., 2021
Real time RPA	<1 h	DNA	39°C, Portable fluorimeter	2.16 pg	Rapidness, portability	Low sensitivity	Lei et al., 2019
RPA/Cas Fluorescence	45 min	DNA	37°C, Portable fluorimeter	0.221 pg	High specificity and sensitivity	Qualitative testing	This study
RPA/Cas LFD	50 min	DNA	37°C in Incubator	0.221 pg	High specificity and sensitivity, portability	Qualitative testing	This study

## Conclusion

In this study, a rapid and sensitive detection method based on RPA/CRISPR/Cas12a system was developed for the diagnosis of aggressive *L. maculans*, which is responsible for blackleg disease in *B. napus*. The limit of detection was down to 4.7 genomic DNA copies, which was 100 times higher than that of fluorescent RPA (Lei et al., 2019) and 10 times higher than that of real-time PCR (Zhou et al., 2011). With portable one-pot detection and AOCLFA, the lyophilized RPA reagents and CRISPR/Cas12a reagents, along with lateral flow strip detection were fulfilled in one tube or one chip, which offered easy storage and transportation of reagents. In the practical application of AOCLFA, a portable incubator pad or box was used to provide the required temperature (37~40°C) for the RPA pre-amplification and Cas12a/crRNA reaction. Furthermore, the minimum equipment (pipettes, reagent tubes, thermal block) and DNA extraction reagents could be contained in a portable suitcase, which is applicable for field diagnosis of phytopathogenic fungi.

## Data availability statement

The original contributions presented in the study are included in the article/Supplementary material, further inquiries can be directed to the corresponding authors.

## Author contributions

RL, YL, and XW designed, conceived, and performed the experiments. RL and XW wrote the manuscript. LL, JW, and LJ validated the experiments. PW, ZC, and XL contributed fungi materials and reviewed the manuscript. All authors read and approved the final manuscript.

## Funding

This work was financially supported by National Key R&D Program of China (2021YFC2600402), Basic Scientific Research Foundation of Chinese Academy of Inspection and Quarantine (2020JK048), and National Natural Science Foundation of China (11702047).

## Conflict of interest

The authors declare that the research was conducted in the absence of any commercial or financial relationships that could be construed as a potential conflict of interest.

## Publisher's note

All claims expressed in this article are solely those of the authors and do not necessarily represent those of their affiliated organizations, or those of the publisher, the editors and the reviewers. Any product that may be evaluated in this article, or claim that may be made by its manufacturer, is not guaranteed or endorsed by the publisher.
