# Supplementary material for: A CRISPR/Cas12a-based portable platform for rapid detection of Leptosphaeria maculans in Brassica crops
Source: Front Plant Sci. 2022 Sep 8;13:976510. doi: 10.3389/fpls.2022.976510 (PMC9493447; doi:10.3389/fpls.2022.976510)
Supplement: Supplementary file 1 [file Data_Sheet_1.docx]

A CRISPR/Cas12a based Portable Platform for Rapid Detection of *Leptosphaeria maculans* in *Brassica* Crops

**Rong Lei^1^, Yuan Li^2^, Limei Li^3,4^, Jingyi Wang^4^, Zhenhai Cui^5^, Rui Ju^4^, Li Jiang^1^, Xiaoling Liao^6*^, Pinshan Wu^1*^ and Xinyi Wang^3*^**

*^1^Chinese Academy of Inspection and Quarantine, Beijing, China, ^2^Central Laboratory of Yongchuan Hospital, Chongqing Medical University, Chongqing, China, ^3^School of Life and Health, Dalian University, Dalian, China, ^4^Shenyang Agricultural University, Shenyang, China, ^5^Key Laboratory of Soybean Molecular Design and Breeding, Northeast Institute of Geography and Agroecology, Chinese Academy of Sciences, Changchun, China, ^6^Chongqing University of Science and Technology, Chongqing, China*

**^*^ Correspondence:** Xinyi Wang [wangxinyi163@163.com](mailto:wangxinyi163@163.com); Pinshan Wu [wupinshan@163.com](mailto:wupinshan@163.com); Xiaoling Liao [zxc_228@163.com](mailto:zxc_228@163.com)

**Supplementary Figure 1**. (A, B) AutoCAD design for all-in-one chip microfluidics; (C) Picture of the all-in-one chip; (D) The DNA solution was located in the RPA chamber; (E) The RPA reaction buffer was located in the CRISPR chamber; (F) The final CRISPR/Cas12a products was located in the detection chamber, where a lateral flow strip was inserted.

**Supplementary Table 1.** Sequences involved in this study

| **Name** | **Sequences (5'-3')** |
| --- | --- |
| Lm-F | CGTAGGTGAACCTGCGGAAGGATCATTACTCA |
| Lm-R | GCAAGTGGTTTTAGGGGATCCAATTGGTGGG |
| Lm-crRNA | UAAUUUCUACUCUUGUAGAUAAAGCACUGCCGCCCCGAUC |
| ssDNA FQ reporter | 6-FAM/ TTATT/BHQ1 |
| ssDNA 5 bp LF reporter | 6-FAM/ TTATT/Biotin |
| ssDNA 20 bp LF reporter | 6-FAM/ TTTTTTTTTTTTTTTTTTTT/Biotin |
| Target dsDNA | CGTAGGTGAACCTGCGGAAGGATCATTACTCATTTTCAAAGCACTGCCGCCCCGATCAGTGGCGGCAGTCTACTTTGATTCTACCCATGTTTTTTGCGTACTATTTGTTTCCTTGGTGGGCTTGCCCACCAATTGGATCCCCTAAAACCACTTGC |
| *L. maculans* ITS gene (NCBI No. M96384.1) | CCTCCGTAGGTGAACCTGCGGAAGGATCATTACTCATTTTCAAAGCACTGCCGCCCCGATCAGTGGCGGCAGTCTACTTTGATTCTACCCATGTTTTTTGCGTACTATTTGTTTCCTTGGTGGGCTTGCCCACCAATTGGATCCCCTAAAACCACTTGCAATTGCAGTCAGCGTCAGTAACAATGTAATAAATTACAACTTTCAACAACGGATCTCTTGGTTCTGGCATCGATGAAGAACGCAGCGAAATGCGATAAGTAGTGTGAATTGCAGAATTCAGTGAATCATCGAATCTTTGAACGCACATTGCGCCCCTTGGTATTCCATGGGGCATGCCTGTTCGAGCGTCATTTGTACCCTCAAGCTCTGCTTGGTGTTGGGTGTTTGTTCCACTTGGGACTCGCCTTGAAACAATTGGCAGCCGGCACATTGGCCTGGAGCGCAGCACATTTTGCGCCTCTTGTCATGGTTGTTGGCATCCATCAAGACACTTTTTAAGCTCTTGACCTCGGATCAGGTAGGGATACCCGCTGAACTTAAGCATATCAATAAGCGGAGG |

crRNA: CRISPR guided-RNA.

**Supplementary Table 2.** Reaction system of RPA.

| Component | Volume to add (μL) |
| --- | --- |
| Primer Free Rehydration buffer | 29.5 |
| Forward Primer (10 μM) | 2.4 |
| Reverse Primer (10 μM) | 2.4 |
| RNase-free water | 8.6 |
| Total volume | 42.9 |

The components listed in Table are mixed in an Eppendorf tube, and 40 μL of master mix is added to a single pellet aliquot in the TwistAmp® basic kit (TwistDx Inc., Cambridge, UK), and is resuspended on ice. Then the entire reconstituted reaction is transferred back to the initial Eppendorf tube. 2.5 μL of 280 mM Magnesium Acetate (from the TwistAmp Basic kit) is added to the reaction, and then briefly vortex and quickly spin down. Aliquots of 10 μL of the reconstituted RPA reaction are prepared in PCR strip tubes, and 1μL of DNA is added to each reaction. Mix the reaction by carefully pipetting up and down. The reaction is run at 37°C is a pre-heated PCR thermocycler or incubator for 10~30 min. After the reaction is finished, the tubes are stored on ice.

**Supplementary Table 3.** Reaction system of RPA/Cas12a-based fluorescent assay

| Component | Volume to add (μL) |
| --- | --- |
| RPA reaction buffer | 2 |
| crRNA (5 μM) | 0.5 |
| RNase inhibitor (40U/μL) | 0.5 |
| DTT (0.1 M) | 0.5 |
| Cas12a (5 μM) | 1 |
| ssDNA FQ reporter (1 μM) | 2 |
| NEBuffer 3.1 (10x) | 2 |
| Nuclease free water | 11.5 |
| Total | 20 |

The fluorescence assay was performed by mixing the above system and then performing a fluorescence assay at 37˚C in a portable fluorimeter, or real time PCR instrument.

**Supplementary Table 4.** Reaction system of RPA/Cas12a-based lateral flow assay

| Component | Volume to add (μL) |
| --- | --- |
| RPA reaction buffer | 2 |
| crRNA (5 μM) | 0.5 |
| RNase inhibitor (40U/μL) | 0.5 |
| DTT (0.1 M) | 0.5 |
| Cas12a (5 μM) | 1 |
| ssDNA LF reporter (10 μM) | 1 |
| NEBuffer 3.1 (10x) | 2 |
| Nuclease free water | 12.5 |
| Total | 20 |

The lateral flow assay was performed by mixing the above components, and the mixture was incubated for 20 min at 37˚C in a portable incubator. After the reaction finished, 80 μL ddH_2_O is added to the mixture, then a lateral flow strip was inserted into the solution. After 3 minutes, the results can be visible to the naked eye.

**Supplementary Table 5.** The sequence of ITS1/ITS4 amplicons from samples

| No. | Sequence | NCBI identification |
| --- | --- | --- |
| 1 | TGGGCTGGGCATCTACCTGATCCGAGGTCAGAGCTAATAAAATATCTTGATGGATGCCAACAATCATAGCAAGAGGCGCAAAATGTGCTGCGCTCCAGGCCAATATGCCGGCTGCCAATTGTTTTCAGGCGAGTCCAGTCTGCGCAAGCACAGAGAACATTCACCCAACACCAAGCAGAGCTTGAGGGTACAAATGACGCTCGAACAGGCATGCCCCATGGAATACCAAGGGGCGCAATGTGCGTTCAAAGATTCGATGATTCACTGAATTCTGCAATTCACACTACTTATCGCATTTCGCTGCGTTCTTCATCGATGCCAGAACCAAGAGATCCGTTGTTGAAAGTTGTAATTATTACATTGTTACTGACGCTGACTGCAATTACAAGTGGTTTGAATTGTCCTTTTGGCAGGCAAGCCCACCAAGGAAACAAATAGTACGCAAAAAACATGGGTAGAATCAGAAAGGAAAGGGACAGAAATTAAGCCAAAGGCTCAACTGCTGACACCAATCCCCTGATAGAAGGGTAATGATCCTTCCGCAGGTTCACCCTACGGAAG | *Leptosphaeria biglobosa* |
| 2 | ATGGATTGGCATCTACTGATCCGAGGTCACAGCTAATATAATATCTTGATGGATGCCAGCAATCATAGGAAGACGTGCAAAATGTGCTGCGCTCCAGGCCAATATGCCGGATGACAATTGTTTTCGAGTTAAGGCAGTCTGCGCACCCACAGAGAACATTCACCCAACACCAAGCAGAGCTTGATGGCACAAATGACGCTCTAACAGGCATGCCCCATGGAATACCAAGGGGCGCAATGTGAGTTCAAAGATTCCATGATTCACTGAATTCTGTAATTCACACTACTTATCGCATTTCGCTGTCTTCTTCATCGATGCCAGAACCAAGAGATCCGTTGTTGAAAGATGAAATTATTACATTGTTACTGACGCTGACTGCAATAACAAGTGGGTTGAATTGCCCTTTTGCCAGGCAAGCCCACCAAGGAAACAAATAGTACGCACAAAACATGGGTACAATCGGAAATGAAAGGGACCGAAATTAAGCCAAAGGCTCAACTGCTGACACCAATCCCCTGACAGAAAAGGTAATGATCCTTCCGCAGGTTCACCCCTACGGAAG | *Leptosphaeria biglobosa* |
| 3 | GACCTACGGATGGATCTACCTGATCCGAGGTCAGAGCTAATAAAATATCTTGATGGATGCCAACAATCATAGCAAGAGGCGCAAAATGTGCTGCGCTCCAGGCCAATATGCCGGCTGCCAATTGTTTTCAGGCGAGTCCAGTCTGCGCAAGCACAGAGAACATTCACCCAACACCAAGCAGAGCTTGAGGGTACAAATGACGCTCGAACAGGCATGCCCCATGGAATACCAAGGGGCGCAATGTGCGTTCAAAGATTCGATGATTCACTGAATTCTGCAATTCACACTACTTATCGCATTTCGCTGCGTTCTTCATCGATGCCAGAACCAAGAGATCCGTTGTTGAAAGTTGTAATTATTACATTGTTACTGACGCTGACTGCAATTACAAGTGGTTTGAATTGTCCTTTTGGCAGGCAAGCCCACCAAGGAAACAAATAGTACGCAAAAAACATGGGTAGAATCAGAAAGGAAAGGGACAGAAATTAAGCCAAAGGCTCAACTGCTGACACCAATCCCCTGATAGAAGGGTAATGATCCTTCCGCAGGTTCACCCTACGGAAG | *Leptosphaeria biglobosa* |
| 4 | GGGGAACCGGGGATCTACCTGATCCGAGGTCAGAGCTTAAAAAGTGTCTTGATGGATGCCAACAACCATGACAAGAGGCGCAAAATGTGCTGCGCTCCAGGCCAATGTGCCGGCTGCCAATTGTTTCAAGGCGAGTCCCAAGTGGAACAAACACCCAACACCAAGCAGAGCTTGAGGGTACAAATGACGCTCGAACAGGCATGCCCCATGGAATACCAAGGGGCGCAATGTGCGTTCAAAGATTCGATGATTCACTGAATTCTGCAATTCACACTACTTATCGCATTTCGCTGCGTTCTTCATCGATGCCAGAACCAAGAGATCCGTTGTTGAAAGTTGTAATTTATTACATTGTTACTGACGCTGACTGCAATTGCAAGTGGTTTTAGGGGATCCAATTGGTGGGCAAGCCCACCAAGGAAACAAATAGTACGCAAAAAACATGGGCAGAATCAAAGTAGACTGCCGCCACTGATCGAGGCGGCAGTGCTTTGAAAATGGGTAATGATCCTTCCGCAGGTTCACCCTACGGAAGAGGATCATTACCTATTTTCAAAGCACTGGGCGCCTCGATCAGTGGCGGCGTCTACTTTGATTCTGCCCATGTTTTTTGCGTACTATTTGTTTCCTTGGTGGGCTTGCCCACCAATTGGATCCCTAAAACCACTTGCAATTGCAGTCAGCGTCAGTAACAATGTAATAAATTACAACTTTCACAACGGATCTCTTGGTTCTGGCATCGATAAAAACGCAGCGAATGCGATAGTAGTGTGAATTGCAGAATTCAGTGAATCATCGAATCTTTGAACGCACATTGCGCCCCTGGGTATTCATGGGGCATGCTGTTTGAGCGTCATTTGTACCCTCCAGCTCTGCTTGGTGTTGGTGTTTGTTCACTTGGGACTCGCCTTGAAACAATTGGCAGCGGCACATTGGTCTGGAGCGCAGCACATTTTGCGCCTCTCTGTCATGGGTGGTTGGCATCATCAGACACTTTTTAAGCTCTGACCTCGGATCAGTAGGGGATAATCG | *Leptosphaeria maculans* |
| 5 | GTGGAACTGGGCATCCTACCTGATCCGAGGTCAGAGCTAATAAAATATCTTGATGGATGCCAACAATCATAGCAAGAGGCGCAAAATGTGCTGCGCTCCAGGCCAATATGCCGGCTGCCAATTGTTTTCAGGCGAGTCCAGTCTGCGCAAGCACAGAGAACATTCACCCAACACCAAGCAGAGCTTGAGGGTACAAATGACGCTCGAACAGGCATGCCCCATGGAATACCAAGGGGCGCAATGTGCGTTCAAAGATTCGATGATTCACTGAATTCTGCAATTCACACTACTTATCGCATTTCGCTGCGTTCTTCATCGATGCCAGAACCAAGAGATCCGTTGTTGAAAGTTGTAATTATTACATTGTTACTGACGCTGACTGCAATTACAAGTGGTTTGAATTGTCCTTTTGGCAGGCAAGCCCACCAAGGAAACAAATAGTACGCAAAAAACATGGGTAGAATCAGAAAGGAAAGGGACAGAAATTAAGCCAAAGGCTCAACTGCTGACACCAATCCCCTGATAGAAGGGTAATGATCCTTCCGCAGGTTCACCCCTACGGAAG | *Leptosphaeria biglobosa* |
| 6 | GTGGACCTGGGCATCCTACCTGATCCGAGGTCAGAGCTAATAAAATATCTTGATGGATGCCAACAATCATAGCAAGAGGCGCAAAATGTGCTGCGCTCCAGGCCAATATGCCGGCTGCCAATTGTTTTCAGGCGAGTCCAGTCTGCGCAAGCACAGAGAACATTCACCCAACACCAAGCAGAGCTTGAGGGTACAAATGACGCTCGAACAGGCATGCCCCATGGAATACCAAGGGGCGCAATGTGCGTTCAAAGATTCGATGATTCACTGAATTCTGCAATTCACACTACTTATCGCATTTCGCTGCGTTCTTCATCGATGCCAGAACCAAGAGATCCGTTGTTGAAAGTTGTAATTATTACATTGTTACTGACGCTGACTGCAATTACAAGTGGTTTGAATTGTCCTTTTGGCAGGCAAGCCCACCAAGGAAACAAATAGTACGCAAAAAACATGGGTAGAATCAGAAAGGAAAGGGACAGAAATTAAGCCAAAGGCTCAACTGCTGACACCAATCCCCTGATAGAAGGGTAATGATCCTTCCGCAGGTTCACCCCTACGGAAG | *Leptosphaeria biglobosa* |
| 7 | GCGGAAGTGGGCATCTACCTGATCCGAGGTCAGAGCTAATAAAATATCTTGATGGATGCCAACAATCATAGCAAGAGGCGCAAAATGTGCTGCGCTCCAGGCCAATATGCCGGCTGCCAATTGTTTTCAGGCGAGTCCAGTCTGCGCAAGCACAGAGAACATTCACCCAACACCAAGCAGAGCTTGAGGGTACAAATGACGCTCGAACAGGCATGCCCCATGGAATACCAAGGGGCGCAATGTGCGTTCAAAGATTCGATGATTCACTGAATTCTGCAATTCACACTACTTATCGCATTTCGCTGCGTTCTTCATCGATGCCAGAACCAAGAGATCCGTTGTTGAAAGTTGTAATTATTACATTGTTACTGACGCTGACTGCAATTACAAGTGGTTTGAATTGTCCTTTTGGCAGGCAAGCCCACCAAGGAAACAAATAGTACGCAAAAAACATGGGTAGAATCAGAAAGGAAAGGGACAGAAATTAAGCCAAAGGCTCAACTGCTGACACCAATCCCCTGATAGAAGGGTAATGATCCTTCCGCAGGTTCACCCTACGGAAG | *Leptosphaeria biglobosa* |
| 8 | GCGGGAGTGGGCATCTACCTGATCCGAGGTCAGAGCTAATAAAATATCTTGATGGATGCCAACAATCATAGCAAGAGGCGCAAAATGTGCTGCGCTCCAGGCCAATATGCCGGCTGCCAATTGTTTTCAGGCGAGTCCAGTCTGCGCAAGCACAGAGAACATTCACCCAACACCAAGCAGAGCTTGAGGGTACAAATGACGCTCGAACAGGCATGCCCCATGGAATACCAAGGGGCGCAATGTGCGTTCAAAGATTCGATGATTCACTGAATTCTGCAATTCACACTACTTATCGCATTTCGCTGCGTTCTTCATCGATGCCAGAACCAAGAGATCCGTTGTTGAAAGTTGTAATTATTACATTGTTACTGACGCTGACTGCAATTACAAGTGGTTTGAATTGTCCTTTTGGCAGGCAAGCCCACCAAGGAAACAAATAGTACGCAAAAAACATGGGTAGAATCAGAAAGGAAAGGGACAGAAATTAAGCCAAAGGCTCAACTGCTGACACCAATCCCCTGATAGAAGGGTAATGATCCTTCCGCAGGTTCACCCCTACGGAAG | *Leptosphaeria biglobosa* |
| 9 | CTGGACTGGGCATCTACCTGATCCGAGGTCAGAGCTAATAAAATATCTTGATGGATGCCAACAATCATAGCAAGAGGCGCAAAATGTGCTGCGCTCCAGGCCAATATGCCGGCTGCCAATTGTTTTCAGGCGAGTCCAGTCTGCGCAAGCACAGAGAACATTCACCCAACACCAAGCAGAGCTTGAGGGTACAAATGACGCTCGAACAGGCATGCCCCATGGAATACCAAGGGGCGCAATGTGCGTTCAAAGATTCGATGATTCACTGAATTCTGCAATTCACACTACTTATCGCATTTCGCTGCGTTCTTCATCGATGCCAGAACCAAGAGATCCGTTGTTGAAAGTTGTAATTATTACATTGTTACTGACGCTGACTGCAATTACAAGTGGTTTGAATTGTCCTTTTGGCAGGCAAGCCCACCAAGGAAACAAATAGTACGCAAAAAACATGGGTAGAATCAGAAAGGAAAGGGACAGAAATTAAGCCAAAGGCTCAACTGCTGACACCAATCCCCTGATAGAAGGGTAATGATCCTTCCGCAGGTTCACCCCTACGGAAG | *Leptosphaeria biglobosa* |
| 10 | GTGGACCGGGCATCTACCTGATCCGAGGTCAGAGCTAATAAAATATCTTGATGGATGCCAACAATCATAGCAAGAGGCGCAAAATGTGCTGCGCTCCAGGCCAATATGCCGGCTGCCAATTGTTTTCAGGCGAGTCCAGTCTGCGCAAGCACAGAGAACATTCACCCAACACCAAGCAGAGCTTGAGGGTACAAATGACGCTCGAACAGGCATGCCCCATGGAATACCAAGGGGCGCAATGTGCGTTCAAAGATTCGATGATTCACTGAATTCTGCAATTCACACTACTTATCGCATTTCGCTGCGTTCTTCATCGATGCCAGAACCAAGAGATCCGTTGTTGAAAGTTGTAATTATTACATTGTTACTGACGCTGACTGCAATTACAAGTGGTTTGAATTGTCCTTTTGGCAGGCAAGCCCACCAAGGAAACAAATAGTACGCAAAAAACATGGGTAGAATCAGAAAGGAAAGGGACAGAAATTAAGCCAAAGGCTCAACTGCTGACACCAATCCCCTGATAGAAGGGTAATGATCCTTCCGCAGGTTCACCCTACGGAAG | *Leptosphaeria biglobosa* |
| 11 | GCGGAATGGGGCATCTACCTGATCCGAGGTCAGAGCTAATAAAATATCTTGATGGATGCCAACAATCATAGCAAGAGGCGCAAAATGTGCTGCGCTCCAGGCCAATATGCCGGCTGCCAATTGTTTTCAGGCGAGTCCAGTCTGCGCAAGCACAGAGAACATTCACCCAACACCAAGCAGAGCTTGAGGGTACAAATGACGCTCGAACAGGCATGCCCCATGGAATACCAAGGGGCGCAATGTGCGTTCAAAGATTCGATGATTCACTGAATTCTGCAATTCACACTACTTATCGCATTTCGCTGCGTTCTTCATCGATGCCAGAACCAAGAGATCCGTTGTTGAAAGTTGTAATTATTACATTGTTACTGACGCTGACTGCAATTACAAGTGGTTTGAATTGTCCTTTTGGCAGGCAAGCCCACCAAGGAAACAAATAGTACGCAAAAAACATGGGTAGAATCAGAAAGGAAAGGGACAGAAATTAAGCCAAAGGCTCAACTGCTGACACCAATCCCCTGATAGAAGGGTAATGATCCTTCCGCAGGTTCACCCTACGGAAG | *Leptosphaeria biglobosa* |
| 12 | GTGGACTGGGGCATCCTACCTGATCCGAGGTCAGAGCTAATAAAATATCTTGATGGATGCCAACAATCATAGCAAGAGGCGCAAAATGTGCTGCGCTCCAGGCCAATATGCCGGCTGCCAATTGTTTTCAGGCGAGTCCAGTCTGCGCAAGCACAGAGAACATTCACCCAACACCAAGCAGAGCTTGAGGGTACAAATGACGCTCGAACAGGCATGCCCCATGGAATACCAAGGGGCGCAATGTGCGTTCAAAGATTCGATGATTCACTGAATTCTGCAATTCACACTACTTATCGCATTTCGCTGCGTTCTTCATCGATGCCAGAACCAAGAGATCCGTTGTTGAAAGTTGTAATTATTACATTGTTACTGACGCTGACTGCAATTACAAGTGGTTTGAATTGTCCTTTTGGCAGGCAAGCCTACCAAGGAAACAAATAGTACGCAAAAAACATGGGTAGAATCAGAAAGGAAAGGGACAGAAATTAAGCCAAAGGCTCAACTGCTGACACCAATCCCCTGATAGAAGGGTAATGATCCTTCCGCAGGTTCACCCCTACGGAAG | *Leptosphaeria biglobosa* |
| 13 | GATGATTGGGCTCTACCTGATCCGAGGTCACCTTAGAAATGGGGTTGTTGACAGGGTAGCCTCCCGAAGACCCTTTAGCGAATAGTTTCCACAACGCTTACGGTACAGAAGACCCAGTGGGGCCATTTGTGGCTTTTGGGGCACCGCCCTGCCCAATGCCAAGCGAGGCTTGAGTGGTGAAATGACTCTCGAACAGGAATGCCCCCCGGAATGCCAGGGGGCCCAATGAGCGTTCGAAGATTCGAGGATTCACTGAATCCTGCAATTCCTGTTACTTATCGTATTTCTCTGTTATCTTCTTCCATTGCATACTCAATAGATCCCGTGACCAAAATATTAATTTATTAATGATTTATACTCTGACTGCCAAGGAACTCAAAATGTTGAATTGTCAACCCGGAGCCCCCGCCCGAACGGAGGGTCCCCGCGCACGCAACAGAGACGGGCAACAAATGGATATGAATAACCAGGTCGAACCCCAGGCGCCCTTGTAATGATCCCTCCTTCCGTTCGCCTACCGAAGGAAG | *Leptosphaeria biglobosa* |
| 14 | GGGAACTGGGATCTACCTGATCCGAGGTCAGAGCTTAAAAAGTGTCTTGATGGATGCCAACAACCATGACAAGAGGCGCAAAATGTGCTGCGCTCCAGGCTAATGTGCCGGCTGCCAATTGTTTCAAGGCGAGTCCCAAGTGGAACAAACACCCAACACCAAGCAGAGCTTGAGGGTACAAATGACGCTCGAACAGGCATGCCCCATGGAATACCAAGGGGCGCAATGTGCGTTCAAAGATTCGATGATTCACTGAATTCTGCAATTCACACTACTTATCGCATTTCGCTGCGTTCTTCATCGATGCCAGAACCAAGAGATCCGTTGTTGAAAGTTGTAATTTATTACATTGTTACTGACGCTGACTGCAATTGCAAGTGGTTTTAGGGGATCCAATTGGTGGGCAAGCCCACCAAGGAAACAAATAGTACGCAAAAAACATGGGCAGAATCAAAGTAGACTGCCGCCACTGATCGAGGCGGCAGTGCTTTGAAAATGGGTAATGATCCTTCCGCAGGTTCACCCTACGGAAGGGGGAGTATTACCCATTTTCAAAGCACTGGCGCCTCGATAGTGGCGGCGTCTACTTTGATTCTGCCCATGTTTTTTGCGGACTATTTGTTTCCTTGGTGGGCTTGCCCACCAATTGGATCCCCTAAAACCACTTGCAATTGCAGTCAGCGTCAGTAACAATGTAATAAATTACAACTTTCACAACGGATCTCTTGGTTCTGGCATCGATAAAAACGCACCGAATGCGATGAGTAGTGTGAATTGCAGAATTCAGTGAATCATCGAATCTTTGAACGCACATTGCGCCCTTGGTATTCATGGGGGCATGCCTGTTCGAGCGTCATTGTACCTCAGCTCTGCTTGGTGTTGGGTGTTTGTTCACTTGGGACTCGCCTGCAACAATTGGCAGCGGCCATTGGGCTGGAGGCAGCAATTTGCGCTCTGGTCATGGGTGTGGCATCCATCAGACCCTTTTAAGCTCTGACCTCCGACAGGAAGGATAGCGCTTAACT | *Leptosphaeria maculans* |
| 15 | GTGGGACGGGCATCTACCTGATCCAGAGGTCAGAGCTTAAAAAGTGTCTTAGATAGGATGCCAACAACCATAGACAAGAGGCGCAAAATGTGCTGCGCTCCAGGCTAATGTGCCGGCTGCCAATTGTTTCAAGGCGAGTCCCAAGTGGAACAAACACCCAACACCAAGCAGAGCTTGAGGGTACAAATGACGCTCGAACAGGCATGCCCCATGGAATACCAAGGGGCGCAATGTGCGTTCAAAGATTCGATGATTCACTGAATTCTGCAATTCACACTACTTATCGCATTTCGCTGCGTTCTTCATCGATGCCAGAACCAAGAGATCCGTTGTTGAAAGTTGTAATTTATTACATTGTTACTGACGCTGACTGCAATTGCAAGTGGTTTTAGGGGATCCAATTGGTGGGCAAGCCCACCAAGGAAACAAATAGTACGCAAAAAACATGGGCAGAATCAAAGTAGACTGCCGCCACTGATCGAGGCGGCAGTGCTTTGAAAATGGGTAATGATCCTTCCGCAGGTTCACCCTACGGAAGGGGATCTTTACCCATTTTCTAAGGCACTGGGCGCCTCGATCAGTGGCGGCAGTCTACTTTGATTCTGCCCATGTTTTTTGCGGACTATTTGTTTCCTTGGTGGGCTTGCCCCCAATTGGATACCCTAAAACCACTTGCAATTGCAGTCAGCGTCAGTAACAATGTAATAAATTACAACTTTCACAACGGATCTCTTGGTTCTGGCATCGATAAGAACGCAGCGAATGCGATGAGTAGTGTGAATTGCAGAATTCAGTGAATCATCGAATCTTTGAACGCACATTGCGCCCTCGGTATTCATGGGGCATGCTGTTCGAGCGTCATTTGTACCTCAGCTCTGCTGGTGTTGGGTGTTTGTTCACTTGGACTCGCTTGAACATTGCAGCGTCATTGCTGGAGCGCAGCAATTTGGCCTCTGTCATGGTGTTGGCATCATCAGAACTTTTAAGTCTTGACTCGCGATCAG | *Leptosphaeria maculans* |
| 16 | ACATCGGGGGATCGCTGACCTGGGGTCGCGTTGAGGACTTTGGGTCATCAAGAGCTTTTGGACCGGAACGTCTGACTATATGACGAGAATTAAATTCACCACCGCATGTCAAGACGCTCCTGACGTCCTTAGCTCGGATTTTGGCCAACCGCGTGCGGTAACACACGGGAGATCAGCTTCCGTCCCATATCCTCGAGAGGATGGGGGGACGACGATTTGTGACACCCAGGCAGACGTGCCCTCGGCCAGAAGGCTTGGGGCGCAACTTGCGTTCAAAGACTCGATGGTTCACGGGATTCTGCAATTCACACCAAGTATCGCATTTCGCTACGTTCTTCATCGATGCGAGAGCCGAGATATCCGTTGCCGAGAGTCGTTTTAGACTTTACATTGCAGCACTGCTTCCGAACAAACACCGTCTCCGGGTTGGCGAAAGCAGGCTGTTTAGTTGCATTTTCCTTGACACTTTTCGTGCCGGGGTTTGGTGATATCCGGAAGCTATGCGTACGATCCAACCAAAACTGAAGTCTTGGCCAAGGATGAACGCATAACCACGGAATCAGCAGGCACAGTAAGAAACCGGCCTACCGAGAGTGATGTTTCATCGTTCTCAGGTCGTTCTGTTTCCAGGGTACGACAATGATCCTTCCGCAGGTCCCCCTTTACGGAAAG | *Brassica napus genome* |
